# Cones and sleeves present good survival and clinical outcome in revision total knee arthroplasty: a meta-analysis

**DOI:** 10.1007/s00167-021-06670-0

**Published:** 2021-08-13

**Authors:** Laura Theresa Fischer, Markus Heinecke, Eric Röhner, Peter Schlattmann, Georg Matziolis

**Affiliations:** 1 Orthopaedic Department Waldkliniken Eisenberg, Orthopaedic Professorship of the University Hospital Jena, Klosterlausnitzer Str. 81, 07607 Eisenberg, Germany; 2 Department of Medical Statistics, Informatics and Data Science, Jena University Hospital Jena, Bach Str. 18, 07743 Jena, Germany

**Keywords:** Revision total knee arthroplasty, Total knee replacement, Bone defects, Cones, Sleeves, Aseptic loosening

## Abstract

**Purpose:**

The fixation of revision total knee arthroplasties (rTKA) tends to be difficult, leading to a reduction in implant survival. One option for achieving a more stable anchorage is to use metaphyseal cones and sleeves. The objective of the present paper is to provide a current comparative meta-analysis on survival and clinical results of cones vs. sleeves, with a differentiation between the short- and long-term outcome.

**Methods:**

A search of the literature was conducted systematically to include original papers from 2010 to June 2021. The following parameters were taken into account: revision for aseptic loosening, revision for any reason, periprosthetic joint infections (PJI), KSS as well as KSFS. Studies with a mean follow-up of at least 60 months were defined to be long-term follow-up studies (LT). All other studies were included in the short-term (ST) study analysis. A pooled incidence was used as a summary statistic using a random intercept logistic regression model.

**Results:**

The present meta-analysis included 43 publications with 3008 rTKA. Of these, 23 publications with 1911 cases were allocated to the sleeve group (SG) and 20 papers with 1097 cases to the cone group (CG). CG showed overall numerically higher complication rates in short- and long-term follow-up, compared with SG. Aseptic loosening occurred at a rate of 0.4% in SG (LT) and 4.1% in CG (LT) (*p* = 0.09). Periprosthetic joint infection (PJI) was more frequent in the cone group (7% in ST and 11.7% in LT) than in the sleeve group (3.4% in ST and 4.9% in LT, *p* = 0.02 both). The total revision rate was 5.5% in SG (LT) and 14.4% in CG (LT) (*p* = 0.12). The clinical scores were also comparable between the two groups. Hinged prothesis were used more frequent in the cone group (ST *p* < 0.001; LT *p* = 0.10), whereas CC type protheses were used more frequently in the sleeve group (ST *p* < 0.001; LT *p* < 0.11).

**Conclusions:**

This meta-analysis takes into account the longest follow-up periods covered to date. Both cones and sleeves represent a reliable fixation method in the case of severe bone loss in rTKA, although the higher rate of PJI after cone fixation remains a source of concern. A metaphyseal fixation of hinged implants should be taken into account.

**Level of evidence:**

II (meta-analysis).

## Introduction

Regardless of the reason for revision, implant survival is reduced after revision total knee arthroplasty (rTKA) [23]. One reason for this is the challenging fixation of the implant in the bone stock, which is compromised both in substance and in density [5, 8, 14, 28, 29].

Implant fixation and defect management are oriented according to the estimated bone defect and bone quality [11, 28]. Various techniques are used to achieve the stable anchorage of a revision implant. Apart from cement, allografts, wedges and stem extensions, cones and sleeves have become increasingly popular over the past few years. This is because an additional metaphyseal implant anchorage is superior to a sole diaphyseal fixation [22].

Cones may be regarded as metaphyseally anchored metallic bone grafts, which enable a cementless fixation over their porous surface. Sleeves also follow the principle of cement-free metaphyseal anchorage. In contrast to cones, sleeves are firmly attached to the prosthesis. However, it also bears the risk of fractures during broaching, which represents the most common intraoperative complication when sleeves are used [17, 24, 31].

In numerous individual studies, excellent implant survival has been shown both for cones and for sleeves after a medium-term follow-up [6, 9, 12, 17, 21, 23, 33]. The few studies with long-term follow-up (> 7 years) [1, 3, 6, 13, 15, 25] could not be taken into account in previous meta-analyses [18, 27, 30, 34]. Nevertheless, there is initial evidence of a drop in survival over long-term follow-up [1].

It is thereby obvious, that the revision reasons differ depending on timepoint of failure [2, 3]. Revisions within the first years after implantation of cones or sleeves may result from failing bony integration or persistence of infection (in septic revisions). In contrast, late revisions may result from aseptic loosening of a primarily integrated implant or new infection. Given a different aetiology of failure, specific information about the short- and long-term outcome of cones and sleeves is missing.

Since all published meta-analyses [18, 27, 30, 34] include studies without differentiation between short- and long-term survival, there is lack of evidence about differing results of cones and sleeves depending on length of follow-up.

The objective of the present paper was therefore to conduct a current comparative meta-analysis on the survival and clinical outcome of cones vs. sleeves. Here, in contrast to previous meta-analyses, short- and long-term (ST vs. LT) follow-up were to be differentiated.

## Methods

### Literature search strategy

**Fig. 1:**
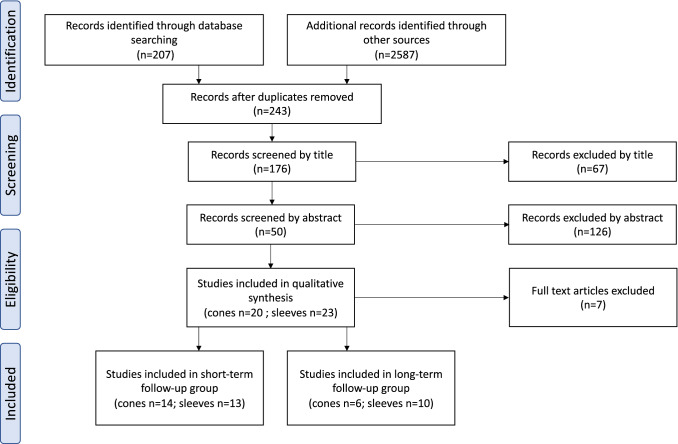
PRISMA flow diagram of article selection. PRISMA—Preferred Reporting Items for Systematic Reviews and Meta-Analysis

The literature search was conducted systematically, following the internationally recognised Preferred Reporting Items for Systematic Reviews and Meta-Analysis (PRISMA) (Fig. 1). The literature databases PubMed, Ovid Medline, GoogleScholar and Cochrane Library were used. The following search terms were used in combination: (“total knee arthroplasty” OR “revision total knee arthroplasty”) AND (“cones” OR “sleeves”).

Original papers published in English between 2010 and June 2021 were included after thorough screening of their relevance with regard to content. All publications with a follow-up of less than 1 year were excluded.

The data were extracted both from the text and from the respective tables and figures. For quality assurance, a second reviewer (M.H.) was consulted in the event of uncertainty and a cross-check was carried out.

For the evaluation of survival, the following parameters were selected: number of implant exchanges in total, implant exchanges due to aseptic loosening and septic reoperations with and without implant exchange e.g. DAIR (debridement, antibiotics and implant retention) in periprosthetic joint infections (PJI). Regarding clinical outcome the KSS (Knee Society Score) and KSFS (Knee Society functional Score) were assessed.

Revisions were defined as implant exchange, excluding PE-exchange only. All complications requiring revision but without prosthesis replacement counted as re-operations.

Descriptive data considered were the mean follow-up of the studies, the reasons for revision (index indication), the level of constraint (non-constrained, condylar constrained (CC) or hinged) as well as the patients’ age and sex.

### Statistics

Prevalence represents the ratio of the number of patients with complications to the total of number of patients in that study. A pooled prevalence was used as a summary statistic using a random intercept logistic regression model. Accordingly, prevalence was used to enable a forest plot. The horizontal bars in the plots represent the range of confidence interval (CI). A 95% CI was used in the analysis. Analysis of heterogeneity of prevalence across studies was initially done using a Chi-square test. The degree of heterogeneity was also quantified using *I*^2^ values. The *I*^2^ statistic describes the percentage of variation across studies that is due to heterogeneity rather than chance. In this meta-analysis the heterogeneity variance tau^2^ was estimated based on the maximum likelihood estimate according to a random effects logistic regression model. Publication bias was investigated using Egger’s regression test [10]. Statistical analysis was performed using the statistical software R using the package meta [4, 32].

## Results

The present meta-analysis included 43 publications with *n* = 3008 rTKA. Of these, 23 publications with 1911 cases were allocated to the sleeve group (SG) and 20 papers with 1097 cases the cone group (CG). In the subgroup analysis according to the respective follow-up period, a total of 27 studies resulted for the ST group and a total of 16 studies were assigned to the LT group. This meta-analysis takes into account the longest follow-up periods covered to date. (Table 1).

**Table 1:** Summary of all studies main results

Author	Year	FU (months)	FU (short term/ long term)	Therapy	patients	knees	Age	BMI	Male	Female	Hinged	CC	Unconstrained	Septic index indication	Aseptic index indication	Revision total	Aseptic loosening	PJI (DAIR and implant exchange)
Abdelaziz	2019	126.5	Long	Cone	25	25	65.0		13	12	25	0	0	0	25	13	10	3
Abdelaziz	2019	49.9	Short	Cone	72	72	70.0	30.0	41	31	72	0	0	72	0	15	7	8
Bohl	2017	40.5	Short	Cone	49	49	69.0	35.7	13	36	5	32	12	12	37	1	1	3
Burastero	2018	43.5	Short	Cone	60	60	67.9	27.7	26	34	18	42	0	60	0	5	3	2
Chalmers	2021	24.0	Short	Cone	163	163	67.0	33.0	75	88	52	106	5	46	117	6	2	16
De Martino	2015	72.0	Long	Cone	18	18	73.0	29.0	6	12	12	6	0	13	5	2	0	2
Derome	2014	33.0	Short	Cone	29	29	70.0		19	10	0	18	11	7	22	1	0	2
Erivan	2020	24.0	Short	Cone	61	61	60.4		30	31	6	12	43	2	59	5	2	8
Girerd	2016	34.0	Short	Cone	51	52	68.0	28.0	25	26	38	14	0	19	33	4	0	4
Hernandez	2021	91.2	Long	Cone	59	62	70.0	34.1	21	38	5	41	16	23	39	18	9	11
Howard	2011	33.0	Short	Cone	24	24	64.0		11	13	10	11	3	7	17	0	0	0
Jensen	2014	47.0	Short	Cone	36	36	69.0		25	11	16	14	6	15	21	4	2	2
Kamath	2015	70.0	Long	Cone	63	66	67.0	33.0	27	36	25	33	8	26	40	3	1	7
Lachiewicz	2013	39.0	Short	Cone	27	27	64.6	35.9	13	14	3	22	2	13	14	2	1	1
Ohlmeier	2020	22.0	Short	Cone	52	52	68.6	30.5	29	23	52	0	0	17	35	2	1	2
Panda	2019	83.0	Long	Cone	59	59	69.7	28.7	18	41			0	26	33	2	0	2
Potter	2016	60.0	Long	Cone	157	157	64.0	32.7	82	75				75	82	27	10	21
Rao	2013	36.0	Short	Cone	26	26	72.0		15	11	26	0	0	7	19	1	0	2
Schmitz	2013	37.0	Short	Cone	38	38	72.0				38	0	0	0	38	2	2	0
Villanueva-Martinez	2013	36.0	Short	Cone	21	21	73.3		7	14	10	11	0	5	16	1	0	2
Agarwal	2018	95.7	Long	Sleeve	103	104	74.7		54	49	0	55	49	31	73	21	7	5
Agarwal	2013	43.0	Short	Sleeve	103	104	69.0		54	49	0	55	49	31	73	2	2	2
Alexander	2013	33.0	Short	Sleeve	28	30	71.0		14	14	0	30	0	8	22	0	0	1
Algarni	2020	49.0	Short	Sleeve	27	27	65.4	37.6	5	22	3	24	0	3	24	1	0	0
Barnett	2014	38.0	Short	Sleeve	34	34	60.0	30.7			3	24	7	9	25	4	2	1
Bloch	2019	91.0	Long	Sleeve	277	319	70.0		133	144	59	260	0	70	249	4	0	4
Bugler	2015	39.0	Short	Sleeve	34	35	72.0	30.2	20	14	0	35	0	0	35	0	0	0
Chalmers	2016	38.0	Short	Sleeve	227	227	66.0	34.0			10	166	51	84	143	8	3	12
Dalury	2016	57.6	Short	Sleeve	40	40	73.0	32.0	19	21	6	34	0	6	34	1	1	0
Fedorka	2016	58.8	Short	Sleeve	50	50	65.6		28	22	0	46	0	25	25	5	3	2
Gill	2020	65.0	Long	Sleeve	31	31					8	18	5			0	0	1
Goettsche	2016	24.0	Short	Sleeve	67	67					0	67	0	16	51	4	1	1
Graichen	2015	43.2	Short	Sleeve	121	121					17	27	77	0	121	12	4	4
Gurel	2021	82.4	Long	Sleeve	30	30	69.9	30.2	10	20	0	30	0	8	22	0	0	0
Huang	2014	29.0	Short	Sleeve	79	83	63.5	33.0	29	50	10	73	0	20	63	6	3	6
Klim	2018	63.6	Long	Sleeve	56	56	73.0	34.0	22	34				56	0	9	0	9
Klim	2020	75.6	Long	Sleeve	93	93	68.0	30.0	39	54	0	93	0	52	41	17	0	15
Lai	2020	24.0	Short	Sleeve	17	17	68.0	30.4	5	12	0	17	0	8	9	0	0	1
Martin-Hernandez	2016	71.5	Long	Sleeve	134	134	75.0	29.8	52	82	0	134	0	12	122	2	0	2
Panesar	2021	91.0	Long	Sleeve	99	99	69.7		46	53	99	0	0	32	67	18	2	11
Stefani	2017	37.0	Short	Sleeve	47	47	71.0				0	47	0	17	30	1	0	1
Watters	2017	63.0	Long	Sleeve	116	116	63.7	30.7	58	58	3	98	13	28	88	3	1	6
Wirries	2019	60.0	Long	Sleeve	47	47	67.2	30.6	8	39	23	24	0	19	28	6	3	3

SG and CG were comparable with regard to age, sex distribution (male/female) and index indication for revision (septic/aseptic). (Tables 2 and 3).

**Table 2:** Patient’s demographics were comparable and without significant differences between the therapy groups

Follow-up	Therapy	Age	BMI	Male (%)	Female (%)
short	cone	67.8	31.5	49.0	51.0
short	sleeve	67.1	33.2	46.0	54.0
long	cone	66.8	32.2	43.8	56.2
long	sleeve	70.2	30.7	44.2	53.4

**Table 3:** The results illustrating the indication for index revision using cone or sleeve, the degree of implant constraint, the rates of implant exchange for any reason, aseptic loosening, and the rates for operative intervention resulting from PJI

short term follow-up (< 5 years)	Cones	Sleeves	*p* value
Aseptic index RTKA	61.2 (27.8–86.5)	80.8 (66.4–90.0)	0.23
Septic index RTKA	38.9 (13.5–72.2)	19.2 (10.0–33.6)	0.23
Hinged implant	68.2 (20.8–94.6)	1.8 (0.4–7.9)	< 0.001
CC implant	20.1 (5.5–52.1)	93.9 (77.0–98.6)	< 0.001
Unconstraint implant	2.0 (0.3–12.2)	0.3 (0.01–9.7)	0.37
Implant exchange for any reason	6.1 (3.9–9.3)	4.5 (2.8–7.1)	0.35
Implant exchange for aseptic loosening	4.3 (2.8–6.3)	2.8 (1.8–4.2)	0.16
PJI (with or without implant exchange)	7.0 (4.8–10.0)	3.4 (2.1–5.4)	0.02

Long-term follow-up (> 5 years)	Cones	Sleeves	*p* value
Aseptic index RTKA	63.9 (39.4–82.8)	60.0 (32.7–82.2)	0.83
Septic index RTKA	36.1 (17.2–60.6)	40.0 (17.8–67.3)	0.83
Hinged implant	64.0 (9.9–96.6)	2.0 (0.02–65.1)	0.10
CC implant	27.7 (5.4–72.1)	89.6 (23.7–99.6)	0.11
Unconstraint implant	1.6 (0.1–31.8)	0.2 (0.2–0.2)	0.23
Implant exchange for any reason	14.4 (5.9–31.0)	5.5 (2.2–12.7)	0.12
Implant exchange for aseptic loosening	4.1 (0.8–19.7)	0.4 (0.1–3.0)	0.09
PJI (with or without implant exchange)	11.7 (8.2–16.3)	4.9 (2.6–9.1)	0.02

Numbers are given in percent with 95% confidence interval in brackets and *p* value for subgroup differences

**Fig. 2:**
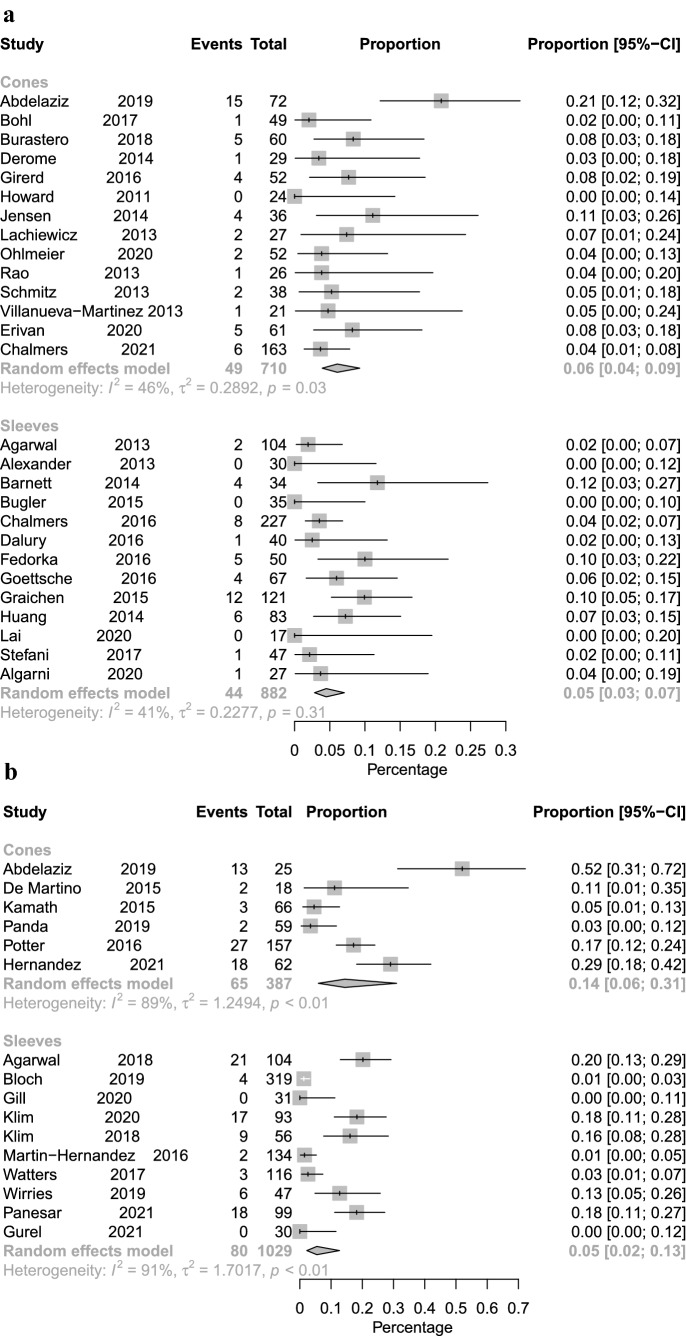
Forest plot illustrating the rates for implant exchange for any reason for cone fixation vs. sleeve fixation with short-term (**A**) and long-term follow-up (**B**)

**Fig. 3:**
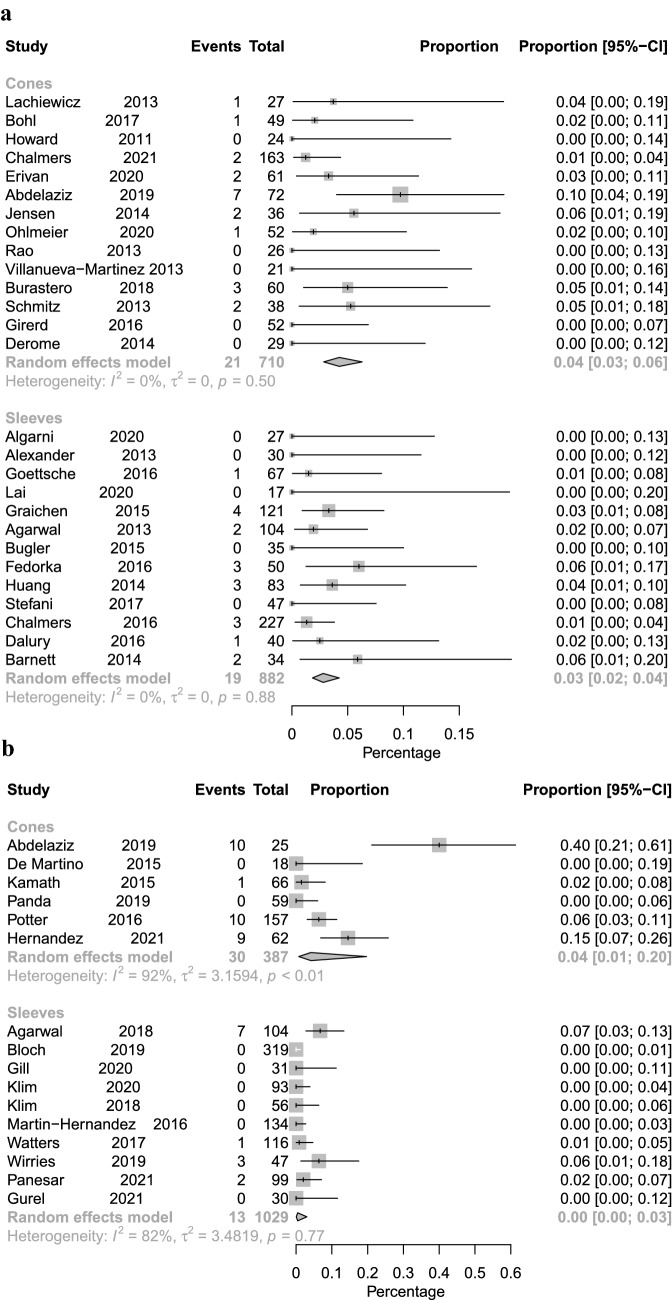
Forest plot illustrating revision rates for aseptic loosening for cone fixation vs. sleeve fixation with short-term (**A**) and long-term follow-up (**B**)

**Fig. 4:**
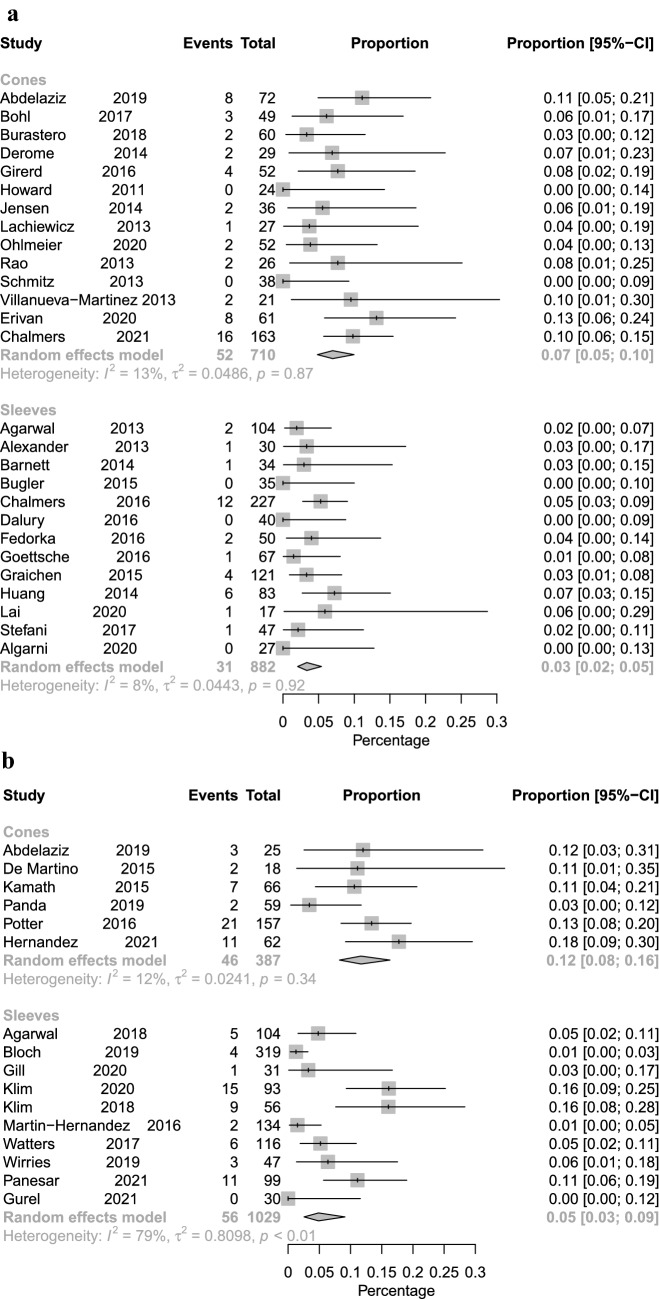
Forest plot illustrating revision for PJI with or without implant exchange for cone fixation vs. sleeve fixation with short-term (**A**) and long-term follow-up (**B**)

With regard to the fixation in groups SG and CG, good short- and long-term prosthesis survival times were seen for both devices. No significant difference was found in relation to prosthesis survival (implant exchange for aseptic loosening, implant exchange for any reason) (Figs. 2 and 3). In contrast to that, periprosthetic joint infections (PJI) were twice frequent in the cone group compared to the sleeve group (Table 3, Fig. 4).

**Fig. 5:**
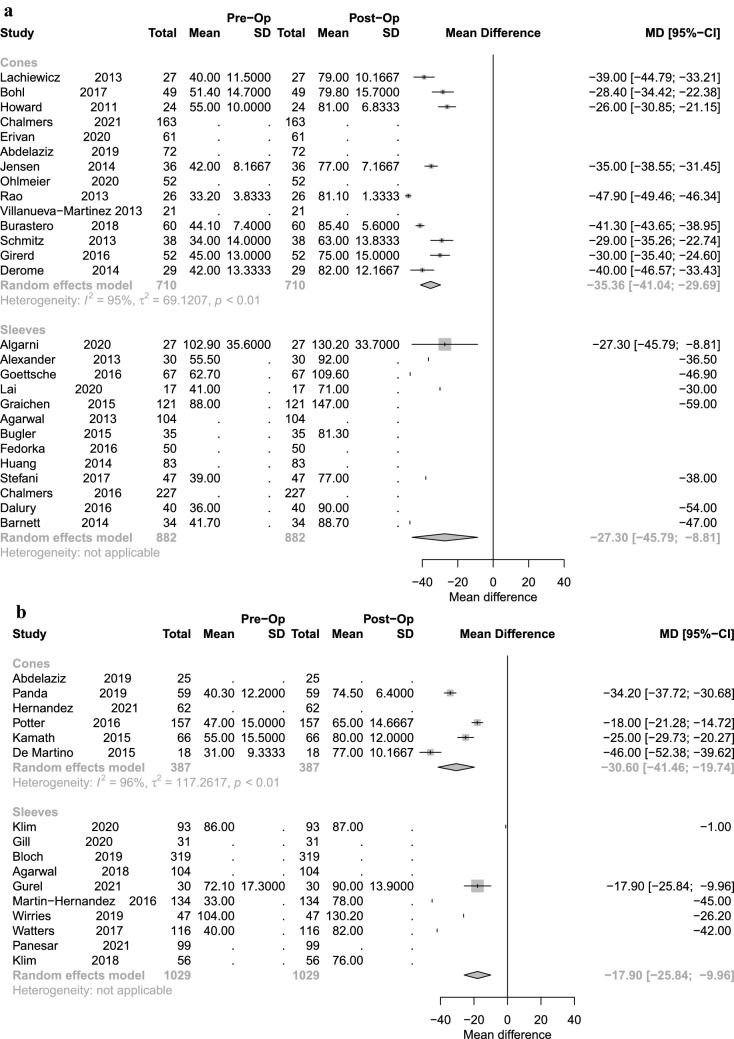
Forest plot illustrating clinical outcome with KSS values in sleeve vs. cone fixation with short-term (**A**) and long-term follow-up (**B**)

**Fig. 6:**
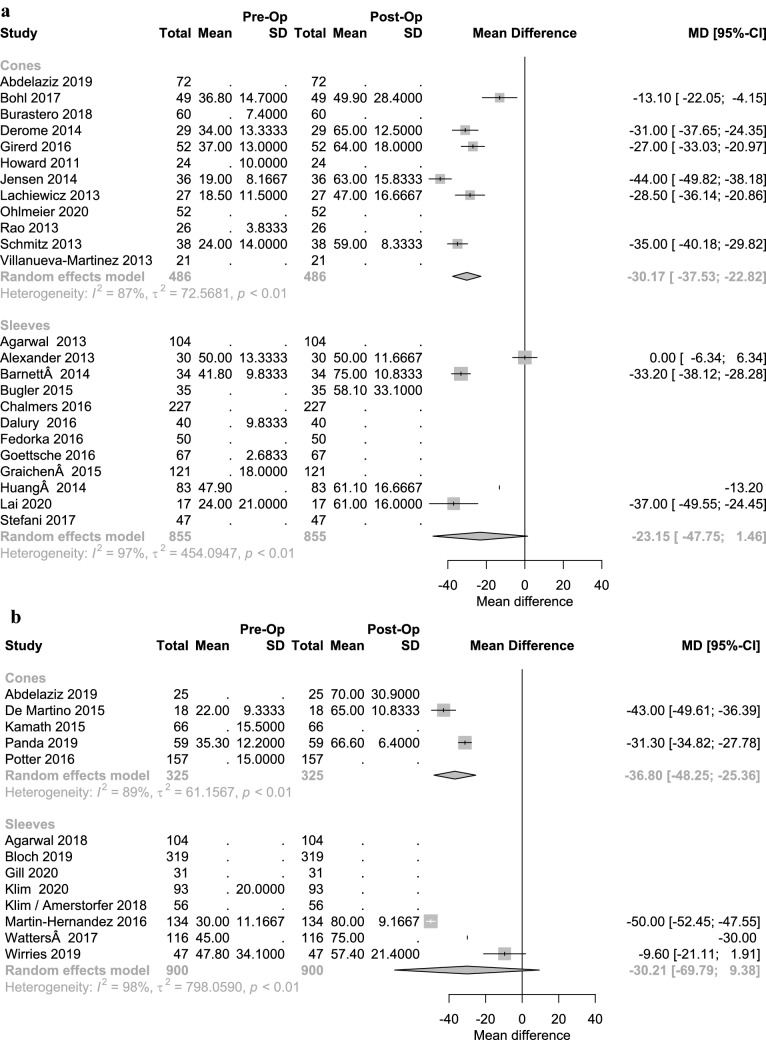
Forest plot illustrating clinical outcome with KSFS values in sleeve vs. cone fixation with short-term (**A**) and long-term follow-up (**B**)

For both devices, significant improvements and good results were seen in all scores (KSS, KSFS) postoperatively (Figs. 5 and 6). A significant superiority of one device over the other could not be demonstrated. Hinged prothesis were used at a higher volume in the CG than in the SG whereas CC type protheses were used more frequently in the SG. (Table 3).

We found publication bias with a bias equal to − 2.86 and *p* value < 0.01. All papers included were level III (retrospective cohort studies, case–control studies) and IV (case series) studies (Table 4).

**Table 4:** Publication bias of the included studies

Author	Year	Therapy	FU (months)	Assembly of comparable groups	Maintenance of comparable groups	High loss to FU (>20%)	Measurements: equal, reliable, valid	Clear definition of interventions	All important outcomes considered	Adjustment for potential confounders	Overall assessed quality
Abdelaziz	2019	Cone	126.5	No	No	Yes	Yes	Yes	No scores	No	Fair
Abdelaziz	2019	Cone	49.9	No	No	Yes	Yes	Yes	No scores	No	Fair
Bohl	2017	Cone	40.5	No	No	No	Yes	Yes	Yes	No	Fair
Burastero	2018	Cone	43.5	No	No	No	Yes	Yes	Yes	No	Fair
Chalmers	2021	Cone	24.0	No	No	No	Yes	Yes	No scores	No	Fair
De Martino	2015	Cone	72.0	No	No	No	Yes	Yes	Yes	No	Fair
Derome	2014	Cone	33.0	No	No	No	Yes	Yes	Yes	No	Fair
Erivan	2020	Cone	24.0	No	No	No	Yes	Yes	No scores	No	Fair
Girerd	2016	Cone	34.0	No	No	No	Yes	Yes	Yes	No	Fair
Hernandez	2021	Cone	91.2	No	No	No	Yes	Yes	No scores	No	Fair
Howard	2011	Cone	33.0	No	No	No	Yes	Yes	Yes	No	Fair
Jensen	2014	Cone	47.0	No	No	No	Yes	Yes	Yes	No	Fair
Kamath	2015	Cone	70.0	No	No	No	Yes	Yes	Yes	No	Fair
Lachiewicz	2013	Cone	39.0	No	No	No	Yes	Yes	Yes	No	Fair
Ohlmeier	2020	Cone	22.0	No	No	No	Yes	Yes	No scores	No	Fair
Panda	2019	Cone	83.0	No	No	No	Yes	Yes	Yes	No	Fair
Potter	2016	Cone	60.0	No	No	No	Yes	Yes	Yes	No	Fair
Rao	2013	Cone	36.0	No	No	No	Yes	Yes	Yes	No	Fair
Schmitz	2013	Cone	37.0	No	No	No	Yes	Yes	Yes	No	Fair
Villanueva-Martinez	2013	Cone	36.0	No	No	No	Yes	Yes	No scores	No	Fair
Agarwal	2018	Sleeve	95.7	No	No	No	Yes	Yes	No scores	No	Fair
Agarwal	2013	Sleeve	43.0	No	No	No	Yes	Yes	No scores	No	Fair
Alexander	2013	Sleeve	33.0	No	No	Yes	Yes	Yes	Yes	No	Fair
Algarni	2020	Sleeve	49.0	No	No	No	Yes	Yes	Yes	No	Fair
Barnett	2014	Sleeve	38.0	No	No	No	Yes	Yes	Yes	No	Fair
Bloch	2019	Sleeve	91.0	No	No	No	Yes	Yes	No scores	No	Fair
Bugler	2015	Sleeve	39.0	No	No	Yes	Yes	Yes	No scores	No	Fair
Chalmers	2016	Sleeve	38.0	No	No	No	Yes	Yes	No scores	No	Fair
Dalury	2016	Sleeve	57.6	No	No	No	Yes	Yes	Yes	No	Fair
Fedorka	2016	Sleeve	58.8	No	No	No	Yes	Yes	No scoreS	No	Fair
Gill	2020	Sleeve	65.0	No	No	No	Yes	Yes	No scores	No	Fair
Goettsche	2016	Sleeve	24.0	No	No	No	Yes	Yes	Yes	No	Fair
Graichen	2015	Sleeve	43.2	No	No	No	Yes	Yes	Yes	No	Fair
Gurel	2021	Sleeve	82.4	No	No	No	Yes	Yes	Yes	No	Fair
Huang	2014	Sleeve	29.0	No	No	No	Yes	Yes	Yes	No	Fair
Klim	2020	Sleeve	63.6	No	No	No	Yes	Yes	Yes	No	Fair
Klim	2018	Sleeve	75.6	No	No	No	Yes	Yes	No scores	No	Fair
Lai	2020	Sleeve	24.0	No	No	No	Yes	Yes	Yes	No	Fair
Martin-Hernandez	2016	Sleeve	71.5	No	No	No	Yes	Yes	Yes	No	Fair
Panesar	2021	Sleeve	91.0	No	No	No	Yes	Yes	No scores	No	Fair
Stefani	2017	Sleeve	37.0	No	No	No	Yes	Yes	Yes	No	Fair
Watters	2017	Sleeve	63.0	No	No	No	Yes	Yes	Yes	No	Fair
Wirries	2019	Sleeve	60.0	No	No	Yes	Yes	Yes	Yes	No	Fair

## Discussion

The most important finding of the present study was that no difference regarding overall prosthesis survival and clinical outcome could be determined neither in short-term nor in long-term follow-up between fixation using cones compared with sleeves in rTKA. The subgroup analyses for the endpoints “implant exchange for aseptic loosening” and “implant exchange for any reason” showed no statistically significant difference, again regardless of the follow-up period. This result has to be interpreted taking into account, that cones were used more frequently with higher constrained implants than sleeves. The endpoint “PJI” differed significantly between the groups presenting a higher infection rate in the cone group.

Available data are predominantly with short follow-up. As a result, previous meta-analyses are biased by a disproportionate evaluation of the short-term follow-up and therefore overestimation of reasons for early revisions. These differ between short- and long-term follow-up [2, 3]. Therefore, studies with short- and long-term follow-up were compared separately in the present meta-analysis.

Bone defects and loss of bone substance are frequently encountered in revision total knee arthroplasty and present a challenge to the surgeon, making the implant fixation complicated. However, an optimal implant fixation is essential for a good functional outcome and survival of the prosthesis. Within the context of rTKA implantations, the epiphysis is almost always damaged and cannot be used as the sole fixation zone. In contrast, the metaphysis is usually sufficiently retained and can be used to anchor the implant [12, 22]. Cones and sleeves are two options available for metaphyseal anchorage.

In knee revision arthroplasty, semi-constrained or hinge prostheses are usually used based on the state of ligaments. Numerous studies have shown that the level of constraint influences the survival time and clinical outcome of the prosthesis. Pure hinge knee prostheses and type 3 bone defects are associated with higher numbers of aseptic loosening and worse clinical outcome [1, 7, 26]. A metaphyseal implant fixation seems to reduce that effect because, despite the significant higher volume of hinged prothesis in the CG, our study shows comparable rates of aseptic loosening between SG and CG. Based on these findings, a metaphyseal fixation of hinged implants should therefore be considered.

Regarding the clinical outcome both devices demonstrated postoperatively significant improvements and good results in all scores (KSS, KSFS) without a significant superiority of one device over the other.

In addition to the aforementioned fixation with cones or sleeves, numerous other factors can influence the subsequent outcome. Levent et al. [20] demonstrated smoking, a large femoral canal anteroposterior diameter and right-sided TKA as significant risk factors for aseptic loosening in TKA. Jasper et al. [16] and Klasan et al. [19] showed younger age, higher knee joint activity and male gender as significant risk factors for repeat revision procedures.

Moreover, both tibial and femoral component can get loose, so that the endpoint loosening is influenced by both components’ fixation. Implant geometry, implantation errors or compromises (rotation, anterior overstuffing, reduction of posterior offset, mediolateral overhang) are more frequent on the femoral than on the tibial side. Therefore, it is to be expected that the femoral component influences the clinical outcome more than the tibial component.

The higher rate of PJI in the CG compared to the SG remains a source of concern and needs further investigation. This cannot be explained by an inclusion bias because the number of septic index operations did not differ between the groups. Apart from generally known reasons for higher rates of postoperative infections that were not systematically assessed by most studies (e.g. smoking, diabetes, malnutrition, obesity, ASA classification), a possible explanation for higher numbers of PJI can be found in the different material properties of cones and sleeves. Sleeves have a dense surface that is structured by surface finish or coating. In contrast, the structure of cones is formed by interconnecting pores, resulting in a large total surface area. Given a relation between foreign material surface area and the risk of late infection this may explain the trend toward more PJI-related revisions in the cone group.

The meta-analyses already published on the clinical and radiological outcome of cones and sleeves have failed to show any statistically significant superiority of one anchorage method over the other. However, they do show a good clinical and radiological outcome for both devices in short- and medium-term follow-up. [18, 27, 30, 34]

There are some limitations to our study. One is the heterogeneous data pool, as not all the papers included in the meta-analysis stated means and standard deviations. The considerable heterogeneity of the data may additionally result from the fact, that revision operations per se are heterogenous (e.g. indication, bone defect, soft tissue situation, number of previous operations). Another limitation is the number of patients. It is a decimal power smaller than in studies dealing with primary TKA, so that few outliers have a higher impact on the given standard deviations. This meta-analysis is further limited by a significant publication bias that could not be eliminated by additional literature data after a second search. Only in very few cases, the bone defects were classified consistently, which means that an indication bias cannot be ruled out. In addition, the surgical technique, implant anchorage (with regard to cementation and/or additional stem anchorage), level of constraint of the implanted prostheses and the definition of complications, re-operations and revisions are not uniform.

According to present knowledge, cones and sleeves have not been directly checked against each other in a randomised controlled trial yet. All results and conclusions of the present meta-analysis must be considered with respect to the quality of the individual studies.

## Conclusion

In conclusion, both cones and sleeves represent a reliable fixation method for revision knee arthroplasty in the case of severe bone loss. Based on our results, we recommend taking an additional metaphyseal fixation of hinged implants into account. While there is no apparent superiority of one method over the other regarding the overall survival and clinical outcome, the higher rate of PJI after cone fixation remains a source of concern.

## References

[1] Abdelaziz H, Jaramillo R, Gehrke T, Ohlmeier M, Citak M (2019). Clinical survivorship of aseptic revision total knee arthroplasty using hinged knees and tantalum cones at minimum 10-year follow-up. J Arthroplasty.

[2] Agarwal S, Azam A, Morgan-Jones R (2013). Metal metaphyseal sleeves in revision total knee replacement. Bone Joint J.

[3] Agarwal S, Neogi DS, Morgan-Jones R (2018). Metaphyseal sleeves in revision total knee arthroplasty: minimum seven-year follow-up study. Knee.

[4] Balduzzi S, Rücker G, Schwarzer G (2019). How to perform a meta-analysis with R: a practical tutorial. Evid Based Ment Health.

[5] Bernatz JT, Brooks AE, Squire MW, Illgen RI, Binkley NC, Anderson PA (2019). Osteoporosis is common and undertreated prior to total joint arthroplasty. J Arthroplasty.

[6] Bloch BV, Shannak OA, Palan J, Phillips JR, James PJ (2020). Metaphyseal sleeves in revision total knee arthroplasty provide reliable fixation and excellent medium to long-term implant survivorship. J Arthroplasty.

[7] Burastero G, Cavagnaro L, Chiarlone F, Alessio-Mazzola M, Carrega G, Felli L (2018). The use of tantalum metaphyseal cones for the management of severe bone defects in septic knee revision. J Arthroplasty.

[8] Chang CB, Kim TK, Kang YG, Seong SC, Kang S-B (2014). Prevalence of osteoporosis in female patients with advanced knee osteoarthritis undergoing total knee arthroplasty. J Kor Med Sci.

[9] De Martino I, De Santis V, Sculco PK, D’Apolito R, Assini JB, Gasparini G (2015). Tantalum cones provide durable mid-term fixation in revision TKA. Clin Orthop Relat Res.

[10] Egger M, Smith GD, Schneider M, Minder C (1997). Bias in meta-analysis detected by a simple, graphical test. BMJ.

[11] Engh GA (2006). Classification of bone defects femur and tibia.

[12] Gill UN, Ahmed N, Noor SS, Memon IA, Memon ZA (2020). Management of the bone loss by metaphyseal sleeves in primary and revision knee arthroplasty: clinical experience and outcome after forty three cases. Int Orthop.

[13] Gurel R, Morgan S, Elbaz E, Ashlenazi I, Snir N, Kadar A (2021). Mid-term clinical and radiographic outcomes of porous-coated metaphyseal sleeves used in revision total knee arthroplasty. Knee Surg Rel Res.

[14] Ha CW, Park YB (2020). Underestimation and undertreatment of osteoporosis in patients awaiting primary total knee arthroplasty. Archiv Orthop Trauma Surg.

[15] Hernandez NM, Hinton ZW, Wu CJ, Ryan SP, Bolognesi MP (2021). Mid-term results of tibial cones : reasonable survivorship but increased failure in those with significant bone loss and prior infection. Bone Joint J.

[16] Jasper L, Jones C, Mollins J, Pohar S, Beaupre L (2016). Risk factors for revision of total knee arthroplasty: a scoping review. BMC Musc Disord.

[17] Kamath AF, Lewallen DG, Hanssen AD (2015). Porous tantalum metaphyseal cones for severe tibial bone loss in revision knee arthroplasty: a five to nine-year follow-up. JBJS.

[18] Kim HJ, Lee O-S, Lee SH, Lee YS (2018). Comparative analysis between cone and sleeve in managing severe bone defect during revision total knee arthroplasty: a systematic review and meta-analysis. J Knee Surg.

[19] Klasan A, Magill P, Frampton C, Zhu M, Young SW (2021). Factors predicting repeat revision and outcome after aseptic revision total knee arthroplasty: results from the New Zealand Joint Registry. Knee Surg Sports Traumatol Arthrosc.

[20] Levent A, Suero EM, Gehrke T, Bakhtiari IG, Citak M (2021). Risk factors for aseptic loosening in complex revision total knee arthroplasty using rotating hinge implants. Int Orthop.

[21] Martin-Hernandez C, Floria-Arnal LJ, Muniesa-Herrero MP, Espallargas-Donate T, Blanco-Llorca JA, Guillen-Soriano M (2017). Mid-term results for metaphyseal sleeves in revision knee surgery. Knee Surg Sports Traumatol Arthrosc.

[22] Morgan-Jones R, Oussedik S, Graichen H, Haddad F (2015). Zonal fixation in revision total knee arthroplasty. Bone Joint J.

[23] Panda I, Wakde O, Singh H, Rajgopal A (2018) Management of large bone defects around the knee using porous tantalum trabecular metal cones during complex primary and revision total knee arthroplasty. Paper presented at: Seminars in Arthroplasty 2018

[24] Panegrossi G, Ceretti M, Papalia M, Casella F, Favetti F, Falez F (2014). Bone loss management in total knee revision surgery. Int Orthop.

[25] Panesar K, Al-Mouazzen L, Nessa L, Jonas SC, Agarwal S, Morgan-Jones R (2021). Revision total knee arthroplasty using an uncemented metaphyseal sleeve, rotating hinge prosthesis: a case series of 99 patients. J Arthroplasty.

[26] Potter GD, Abdel MP, Lewallen DG, Hanssen AD (2016). Midterm results of porous tantalum femoral cones in revision total knee arthroplasty. JBJS.

[27] Roach RP, Clair AJ, Behery OA, Thakkar SC, Iorio R, Deshmukh AJ (2020). Aseptic loosening of porous metaphyseal sleeves and tantalum cones in revision total knee arthroplasty: a systematic review. J Knee Surg.

[28] Rosso F, Cottino U, Dettoni F, Bruzzone M, Bonasia DE, Rossi R (2019). Revision total knee arthroplasty (TKA): mid-term outcomes and bone loss/quality evaluation and treatment. J Orthop Surg Res.

[29] Russell LA (2013). Osteoporosis and orthopedic surgery: effect of bone health on total joint arthroplasty outcome. Curr Rheumatol Reports.

[30] Scott Kelly L., Abdel Matthew P., Hanssen Arlen D., Bono JV, Scott RD (2018). Metaphyseal sleeves and cones in revision total knee arthroplasty. Revision total knee arthroplasty.

[31] Sculco PK, Abdel MP, Hanssen AD, Lewallen DG (2016). The management of bone loss in revision total knee arthroplasty: rebuild, reinforce, and augment. Bone Joint J.

[32] Team RC (2013) R: a language and environment for statistical computing

[33] Watters TS, Martin JR, Levy DL, Yang CC, Kim RH, Dennis DA (2017). Porous-coated metaphyseal sleeves for severe femoral and tibial bone loss in revision TKA. J Arthroplasty.

[34] Zanirato A, Formica M, Cavagnaro L, Divano S, Burastero G, Felli L (2019). Metaphyseal cones and sleeves in revision total knee arthroplasty: two sides of the same coin? Complications, clinical and radiological results-a systematic review of the literature. Musculoskelet Surg.

